# Not Everything Is as It Seems: A Case Series and Overview of Diseases Mimicking Antineutrophil Cytoplasmic Antibody-Associated Vasculitis

**DOI:** 10.3390/jcm12196144

**Published:** 2023-09-23

**Authors:** Eline Houben, Pieter F. de Groot, Yosta Vegting, Josephine M. I. Vos, Erfan Nur, Marc L. Hilhorst, A. E. (Liesbeth) Hak, Arjan J. Kwakernaak

**Affiliations:** 1 Department of Medicine, Division of Clinical Immunology and Allergy, Vasculitis Center of Expertise, Amsterdam University Medical Center, University of Amsterdam, 1105 AZ Amsterdam, The Netherlands; e.houben1@amsterdamumc.nl (E.H.);; 2 Amsterdam Institute for Infection and Immunity, Amsterdam University Medical Center, University of Amsterdam, 1105 AZ Amsterdam, The Netherlands; 3 Department of Medicine, Division of Nephrology, Amsterdam University Medical Center, University of Amsterdam, 1105 AZ Amsterdam, The Netherlands; 4 Department of Hematology, Cancer Center Amsterdam, Amsterdam University Medical Center, University of Amsterdam, 1105 AZ Amsterdam, The Netherlands

**Keywords:** ANCA-associated vasculitis, mimicking disease, mycobacterium avium complex, cryoglobulinemic vasculitis, NK/T-cell lymphoma

## Abstract

Antineutrophil cytoplasmic antibody (ANCA)-associated vasculitis (AAV) is a rare heterogeneous disease in which treatment must be initiated early to prevent irreversible organ damage and death. There are several diseases that can mimic AAV, even in the presence of positive ANCA serology and/or histological evidence of vasculitis, as demonstrated in this case series. We reflect on the diagnostic approach of patients with AAV and provide an overview of AAV-mimicking diseases that can be considered in patients with atypical disease presentation or course.

## 1. Introduction

Antineutrophil cytoplasmic antibody (ANCA)-associated vasculitis (AAV) is a rare and heterogeneous disease causing inflammation of the blood vessel wall of small- (and medium-) sized blood vessels. AAV can be classified clinically into granulomatosis with polyangiitis (GPA), microscopic polyangiitis (MPA) and eosinophilic granulomatosis with polyangiitis (eGPA) and is often associated with antibodies directed against proteinase-3 (PR3) or myeloperoxidase (MPO) [1,2]. Without treatment the disease usually progresses rapidly, resulting in irreversible organ damage and death [3]. Diagnosing AAV may be straightforward in patients with typical clinical features, such as a pulmonary-renal syndrome in the presence of ANCA and/or histological confirmation of small-vessel vasculitis. However, establishing the diagnosis can be challenging in patients with atypical and/or rare disease manifestations, for instance cerebral vasculitis, gynecological or myocardial involvement, as we have encountered in our clinic and has been reported by others [4,5,6]. Positive ANCA serology or histologic confirmation in these cases are considered essential. 

In contrast, some patients present with clinical features consistent with AAV but turn out to have had an alternative disease that mimicked AAV, even in the presence of positive ANCA serology and/or histological evidence of vasculitis, as we present in this case series. The challenging task for clinicians is to decide whether the diagnosis of AAV is appropriate and to keep in mind the urgent time frame in order to prevent irreversible organ damage. Upfront knowledge of differential diagnoses of AAV and its mimicking diseases is of great value. In the current paper we present three cases with AAV-mimicking diseases. Furthermore, we reflect on the diagnostic approach in patients with AAV and provide an overview of AAV-mimicking diseases.

## 2. Case Series

**Figure 1:**
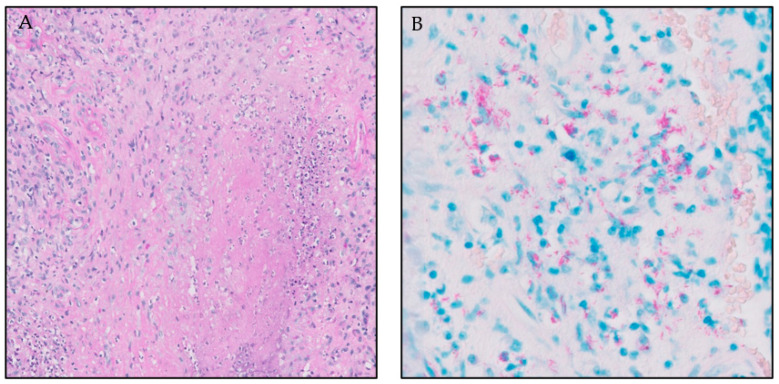
(**A**). Nasal mucosal biopsy with a dense lymphocytic infiltrate and necrosis. (**B**). Nasal mucosal biopsy with positive Ziehl–Neelsen staining.

Case 1: A 68-year-old woman presented with saddle nose deformity, unilateral bloody nasal discharge and crusting. Her past medical history was unremarkable. Laboratory investigation showed increased inflammatory markers, normocytic anemia and normal kidney function without proteinuria or erythrocyturia. Upon rhinoscopy, purulent crusting and rapidly bleeding mucosa were noted, and a computed tomography (CT) scan of the sinuses showed opacification and bone destruction surrounding the left and right orbital wall and the left maxillary sinus. A nasal mucosal biopsy showed multiple areas of granulomatous inflammation with infiltration of lymphocytes, plasma cells, histiocytes and eosinophils and a lymphocytic infiltrate in the artery walls consistent with vasculitis. There was no storiform fibrosis or obliterative phlebitis. The periodic acid–Schiff (PAS), Grocott stain, and polymerase chain reaction (PCR) for mycobacterium tuberculosis were negative. No pulmonary or neurologic abnormalities were present. Anti-PR3 and anti-MPO antibodies (enzyme-linked immunosorbent assay/ELISA) were negative. In line with international consensus [7], we performed an alternative ANCA test (immunoblot), because of the high clinical suspicion of AAV which turned out positive for anti-PR3. We had no clinical suspicion of cocaine abuse. Based on the clinical presentation, histological confirmation of vasculitis and positive PR3-ANCA immunoblot, we diagnosed the patient with GPA with isolated ear–nose–throat (ENT) involvement and bone destruction. We started remission induction treatment with glucocorticoids and rituximab, followed by glucocorticoid tapering and rituximab maintenance. During the following year, inflammatory markers normalized and PR3-ANCA became undetectable using the immunoblot; however, the patient suffered from ongoing nasal discharge with crust formation and pain. Rhinoscopic re-evaluation showed substantial ongoing inflammation and a new nasal mucosal biopsy demonstrated a dense lymphocytic infiltrate with necrosis and giant cells, which guided us again to mycobacterial testing. Ziehl–Neelsen staining, PCR and culture for mycobacterium avium complex (MAC) turned out positive (Figure 1A,B). On revision, a PCR for MAC on the initial nasal mucosal biopsies (taken before the start of treatment) turned out positive as well. It is noteworthy that the PCR for mycobacterium performed at initial presentation was negative because it detects only tuberculous and not non-tuberculous mycobacteria. We concluded that the patient suffered from a chronic nasal infection caused by MAC. Immunosuppressive therapy was stopped and antimicrobial therapy with azithromycin, rifampicin and ethambutol was started, leading to gradual improvement. After ruling out an underlying cellular immunodeficiency (HIV negative, normal CD3+, CD4+ and CD8+ lymphocyte counts and lymphocyte maturation) we explained the MAC infection in this immunocompetent patient by the presence of an oral–nasal fistula due to a previous tooth extraction several years earlier [8]. 

**Figure 2:**
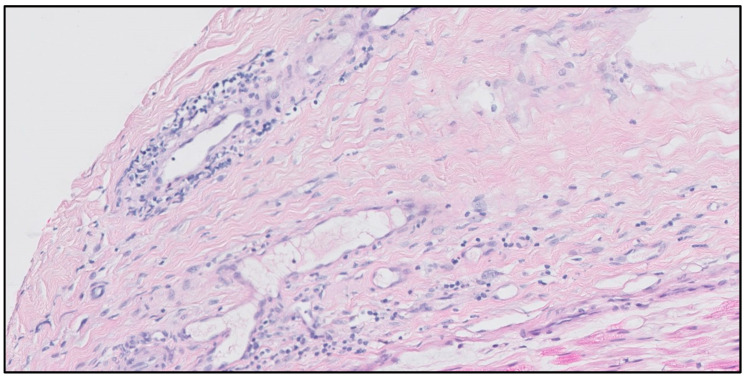
Nerve biopsy demonstrating a lymphocytic infiltrate of the artery wall.

Case 2: A 73-year-old woman was referred to our outpatient clinic because of relapsing ANCA-negative small-vessel vasculitis. Her original presentation consisted of constitutional symptoms, systemic inflammation, normocytic anemia and painful sensorimotor symptoms caused by multiple mononeuropathy of the left and right peroneal nerves and the left ulnar nerve. The multiple mononeuropathy was substantiated by typical EMG abnormalities and a suralis nerve biopsy demonstrating lymphocytic vasculitis (Figure 2).

Serological tests for PR3- and MPO-ANCA were negative. No ENT, pulmonary or renal involvement was present. At this first presentation, the patient had received remission induction treatment with high-dose glucocorticoids and cyclophosphamide, followed by glucocorticoid tapering and azathioprine maintenance. One year later, at referral to our hospital, she again experienced constitutional symptoms and pain of the lower extremities while still on azathioprine maintenance. Systemic inflammation, normocytic anemia, purpura over the lower extremities and a painful sensorimotor neuropathy of the plantar nerve was observed upon clinical evaluation, suggesting disease recurrence. Again, there were no signs of ENT, pulmonary or renal involvement and ANCA serology was negative using two different testing methods (ELISA and immunoblot). We performed the work up for ANCA-negative small-vessel vasculitis and found circulating cryoglobulins (type 2, monoclonal IgM with rheumatoid factor activity, concentration 0.3 g/L). In absence of a cryoglobulin-associated autoimmune disease or infection, we suspected an underlying hematologic malignancy and performed a bone marrow biopsy showing a monoclonal B-cell population, classified as marginal-zone lymphoma. No lymphadenopathy or hepatosplenomegaly were present in a neck/thorax/abdomen CT-scan. We revised the nerve biopsy taken one year earlier and found that the lymphocytic infiltrate was of monoclonal origin with a molecular make up consistent with the monoclonal B-cell population currently found in the bone marrow. We diagnosed the patient with indolent extra-nodal marginal-zone lymphoma with infiltration of the peripheral nervous system and type 2 cryoglobulinemia. Treatment with rituximab and glucocorticoids resulted in an excellent clinical and biochemical response. The cryoglobulins were no longer detectable during follow-up visits. 

**Figure 3:**
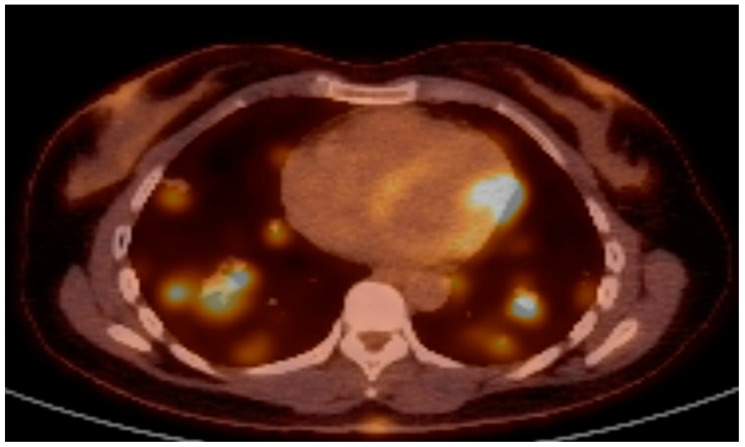
PET-CT scan demonstrating FDG-avid lesions in heart and lungs.

Case 3: A 43-year-old Moroccan woman presented with pain and redness of both eyes accompanied by blurred vision, constitutional symptoms, shortness of breath and numbness of her right upper arm. On physical examination, mild sensorimotor loss of the right arm and the left foot and skin ulcerations were noted. Laboratory investigation showed low inflammatory markers, dysmorphic erythrocyturia and non-nephrotic proteinuria with normal kidney function. Ophthalmologic evaluation demonstrated bilateral panuveitis. A positron emission tomography (PET)-CT scan revealed pulmonary nodules exhibiting ground glass opacities and multiple hypodense fluorodeoxyglucose (FDG)-avid lesions in the left ventricular myocardium (Figure 3). In light of multi-organ involvement including pulmonary, renal and neurologic manifestations, AAV was among the suspected diagnoses, although panuveitis is atypical and ANCA testing yielded negative results. The infectious disease work up was negative. Other differential considerations included Behçet’s disease (upon request she reported oral canker sores), sarcoidosis (with ocular, pulmonary and myocardial involvement) and hematologic malignancy although no lymphadenopathy was present. 

The patient developed weakness of her right arm and left foot and facial paralysis shortly after the initial presentation. A CT scan of the head showed an irregular mass, predominantly on the right side of the brain. Histological examination of a pulmonary lesion revealed an NK/T-cell lymphoma upon which chemotherapy was started. The patients’ clinical course was further complicated by intracranial hemorrhage, originating from the intracerebral lesion and disease progression. She subsequently declined further diagnostic investigation and treatment and died shortly thereafter.

## 3. Discussion

We present three cases in which the original diagnosis of AAV was revised at different time points in the disease course. Although the clinical presentations of these cases were very diverse, several overarching lessons can be learned. We will discuss these lessons and provide a framework for the consideration of AAV mimickers in the diagnostic process of AAV.

In our first patient with MAC infection, retrospectively, several clues suggested an AAV-mimicking disease even before the inadequate response to remission induction treatment. First, her nasal symptoms were mostly unilateral; this is unusual in AAV. Second, the lymphocytic infiltration of the artery wall was relatively modest in respect to the large area of mucosal inflammation, suggesting that the vasculitis was secondary rather than primary. The fact that the second ANCA test came back positive led to early closure of our clinical reasoning. The association between positive ANCA serology and infections such as tuberculosis and MAC has been described previously [9,10]. However, it is not clear whether the presence of ANCA truly reflects a vasculitis induced by mycobacteria or merely a clinical non-significant antibody induced by the infection. One could argue that it is remarkable that the patient did not deteriorate due to the spreading of the MAC infection during immunosuppressive therapy. However, MAC is a slow-growing bacteria and there is no impact of anti-CD20 therapy on the host defense against intracellular bacteria. Absence of clinical improvement after remission induction therapy, which is generally highly effective in AAV, triggered us to re-evaluate the initial diagnosis.

The second patient presented with a multiple mononeuropathy initially attributed to an ANCA-negative small-vessel vasculitis, as demonstrated by the lymphocytic infiltration of a nerve. Although ANCA is negative in approximately 10% of AAV patients [11], the negative ANCA in combination with the solitary organ involvement and lack of efficacy of maintenance treatment urged us to explore differential diagnoses. Although the detection of circulating cryoglobulins led us to the correct diagnosis, the cryoglobulin itself was not the cause of the neurologic symptoms as demonstrated by the infiltration of the nerve by monoclonal B-lymphocytes. It is intriguing that we found a type 2 cryoglobulin, as one would expect a monoclonal type 1 cryoglobulin in the setting of lymphoma. Cryoglobulinemic vasculitis is an immune-complex mediated disease that activates complement, thus resulting in an inflammatory response in contrast to AAV, in which a neutrophil-induced inflammatory response is targeted against the endothelium with relatively minimal complement deposition [1]. Hence, pronounced complement deposition in a biopsy specimen is more consistent with cryoglobulinemic vasculitis than AAV. Of note, cryoglobulins can only be indirectly detected in pathology specimens by demonstrating PAS-staining positive pseudo thrombi [12]. When an AAV-mimicking disease is suspected, the histology review should zoom in on small malignant clones using molecular techniques to increase sensitivity [13]. It is a valuable lesson that even “histologic proof of vasculitis” should not result in premature closure for the clinician. Careful review of the histology report and discussion with the pathologist in the context of the clinical presentation of the patient are of great importance.

The third patient presented with neurological, pulmonary, renal, ocular and skin involvement, which led to the suspicion of systemic vasculitis. However, bilateral panuveitis is not a feature of AAV and the ANCA test was negative. Common ocular manifestations of AAV are episcleritis and scleritis [14]. The patient was diagnosed with NK/T-cell lymphoma, which is a rare type of lymphoma that can occur in extra-nodal sites and commonly involves the nasal and oral cavity. These ENT locations can lead to local inflammation, tissue destruction and epistaxis, which share great similarities with ENT involvement in AAV [15]. Our patient with advanced-stage NK/T-cell lymphoma presented with a rapidly progressive disease with multi-organ involvement sharing similarities with rapidly progressive AAV.

In the evaluation of a patient with suspected vasculitis, a clinician should consider primary vasculitides, such as AAV, but also vasculitis secondary to a systemic auto-immune or auto-inflammatory disease (e.g., systemic lupus erythematosus or sarcoidosis) and vasculitis-mimicking diseases. Of note, AAV classification criteria are developed to provide homogeneous patient populations for research settings. Therefore, classification criteria are specific; however, their sensitivity is not adequate to serve as diagnostic criteria for individual patients [16,17,18]. Features that warrant a reassessment of a diagnosis of AAV are, among others, atypical symptoms or disease presentation, treatment refractoriness, ANCA negativity and absence of histological evidence of vasculitis. AAV can present with a high variety of symptoms in virtually every organ system, including symptoms affecting the ENT, lungs, eyes, kidneys, joints, skin and nervous system. These different clinical phenotypes come with different differential diagnoses. For example, a patient presenting with cavitating lung lesions may have tuberculosis or a patient presenting with skin lesions and glomerulonephritis may have infectious endocarditis [19]. Also, antiphospholipid syndrome can present with overlapping clinical features. Cardiac myxoma, a benign tumor that arises in the heart, can cause constitutional symptoms but also embolic phenomena leading to renal damage [20]. Vacuoles, e1 enzyme, x-linked, autoinflammatory, somatic (VEXAS) syndrome should be emphasized here, as it is a mimicker of multiple diseases, including small-vessel vasculitis, and it is relative refractory to standard immunosuppressive treatment [21]. Polyarteriitis nodosa (PAN) should be considered in patients with serology-negative small- or medium-vessel vasculitis, because PAN favors treatment of cyclophosphamide over rituximab and it has a far lower relapse rate compared to AAV. Furthermore, it is crucial that malignancies including lymphomas are not overlooked in the diagnostic work-up. An overview of vasculitis mimickers is provided in Table 1. This table is not exhaustive, as AAV has a broad spectrum of symptoms and different presenting symptoms and therefore diagnostic considerations. 

While ANCA is the hallmark of AAV, its sensitivity and specificity are limited. ANCA can be positive in a variety of conditions such as infection, monoclonal gammopathy and medication, but also in healthy individuals [22,23,24,25]. Given the potential for false-negative ANCA results, the international consensus recommends a second ANCA test; this improves sensitivity but may lead to a less specific outcome, which is reflected in the first case in which, in retrospect, the anti-PR3 test is considered false positive.

In summary, we reported three cases that were considered to have ANCA-associated vasculitis but turned out to have mimicking diseases. Awareness of AAV mimickers can help in preventing early closure in clinical reasoning. AAV mimickers should be considered in patients with an atypical disease presentation or course, as well as in patients not responding adequate to immunosuppressive therapy, even when positive ANCA serology and histological proof of vasculitis exists.

## Figures and Tables

**Table 1:** Overview of AAV-mimicking diseases.

AAV-Mimicking Disease	Overlapping Features with AAV (Besides Constitutional Symptoms, Systemic Inflammation and/or Anemia)	Distinguishing Features from AAV
**Primary small- (and medium-) vessel vasculitis**		
Cryoglobulinemic vasculitis	Skin, nerve and kidney involvement, sometimes artralgia	No eye involvement, rarely lung involvement. Vasomotor symptoms, circulating cryoglobulines, decreased c4, PAS-positive pseudothrombi and/or complement deposition in histology
Polyarteriitis nodosa	Skin, nerve and kidney involvement	No active urinary sediment, negative serology. Abdominal and/or testicular pain, involvement of medium-sized vessels in histology or imaging (corkscrew phenomenon)
Goodpasture syndrome	Kidney and lung involvement	Soley kidney and lung involvement. Positive anti-GBM antibodies (can be false-negative and overlap with AAV exists, i.e., double positivity)
IgA vasculitis	Skin, kidney and joint involvement	No neurological involvement. Restricted IgA deposition in histology using immunofluorescence
Hypocomplementary urticarial vasculitis (syndrome)	Skin, kidney and joint involvement	Clinical urticarial skin lesions. Abdominal symptoms, decreased c3/c4 and anti-c1q antibodies can be present
Behcet’s disease	Small-vessel involvment	Large-vessels involvement, both arterial and venous. Oralgenital ulceration, pseudofolliculitis and uveitis
**Vasculitis in setting of a systemic auto-immune or auto-inflammatory disease**		
IgG4-related disease	Kidney involvement, pseudotumor orbita and pachymeningitis. Note: overlap with AAV exists	No true granulomatous inflammation, granuloma or necrotizing vasculitis. Salivary gland involvement, pancreatitis, retroperitoneal fibrosis, increased serum IgG4, histologic evidence of IgG4-rich lymphoplasma-cytic infiltrate, storiform fibrosis and obliterative flebitis
Systemic lupus erythematosus	Vasculitic skin lesions and kidney involvement	Mostly normal or modest increased inflammatory markers. Vasculitic lung involvement rare. Presence of SLE-specific clinical and laboratory features (e.g., ANA, anti-dsDNA, decreased c3/c4, hemolysis, thrombocytopenia, etc.)
Sjogren’s disease	Vasculitic skin involvement, nerve and kidney involvement	Vasculitic lung involvement rare. Salivary gland involvement, decreased c3/c4 and IgM-RF
Sarcoidosis	Skin, lung, kidney and joint involvement	Uveitis, myocardial involvement and hypercalciaemia. Histologic evidence of non-caseating granulomas
VEXAS syndrome	Scleritis, tracheal involvement and nephritis reported	Macrocytic anemia, vacuolisation of myeloid lineage in bone marrow and UBA1 mutation
**Other diseases**		
**Infection**		
Sepsis with multiorgan failure	Lung infiltrates and kidney involvement	Positive blood cultures and improvement with antibiotic treatement
Endocarditis	Vasculo-occlussive lesions and kidney involvement	Positive blood cultures or infectious disease serology and echocardiographic heart-valve abnormalities
Tuberculous mycobacterial infection	(Cavitating) lung involvement	Positive Ziehl–Neelsen staining or PCR for tuberculous mycobacteria
Non-tuberculous mycobacterial infection	Sinonasal crusting and discharge and sinonasal bone destruction	Positive Ziehl–Neelsen staining or PCR for non-tuberculous mycobacteria
**Malignancy**		
Solid malignancies, such as lung cancer and gynecological cancer	Depends on the organ affected by the malignancy	Imaging and histopathological investigation
Wide range of different lymphomas	Occasionally lung or kidney involvement	Imaging and histopathological investigation and immunophenotyping and molecular analysis
**Miscellaneous**		
Cocaine abuse	ENT symptoms including nasal septum perforation	History of cocaine use, positive drug screen, often a p-ANCA pattern in indirect immunofluorescence (mostly associated with positive MPO-ANCA in ELISA) and positive anti-elastase antibodies
Thrombotic microangiopathy	Nerve and kidney involvement	Trombocytopenia, Coombs-negatieve hemolysis with fragmentocytes
Cardiac myxoma	Vascular occlusions	Echocardiography of the heart
Calciphylaxis	Skin ulcerations, livedo and necrosis	Occuring in patients with renal replacement therapy, severe pain, livedo localised predominantly on abdominal skin and genitals and calciumphosphate depositions in biopsy
Antiphospholipid syndrome	Purpura, livedo and vasculo-occlusive lesions	Positive antiphospholipid antibodies (note: absence does not rule out seronegative (catastrophic) antiphospholipid syndrome

Abbreviations: AAV: ANCA-associated vasculitis (AAV); c1q: complement 1q; c3: complement 3; c4: complement 4; dsDNA: double-stranded deoxyribonucleic acid; ELISA: enzyme-linked immunosorbent assay; ENT: ear, nose and throat; GBM: glomerular basal membrane; IgA: immunoglobulin A; IgG4: immunoglobulin G4; IgM-RF; immunoglobulin M–rheumatoid factor; MPO: myeloperoxidase; PAS: periodic acid–Schiff; PCR: polymerase chain reaction; PR3: proteinase 3; UBA1: ubiquitin-like modifier activating enzyme 1 and VEXAS: vacuoles, e1 enzyme, x-linked, autoinflammatory and somatic.

## Data Availability

Not applicable.
